# Supplementary figures and images for: Angiotensin II promotes podocyte injury by activating Arf6-Erk1/2-Nox4 signaling pathway
Source: PLoS One. 2020 Mar 2;15(3):e0229747. doi: 10.1371/journal.pone.0229747 (PMC7051060; doi:10.1371/journal.pone.0229747)

## Supporting Information

Supplementary Fig 1. Full length blots of Fig 1C

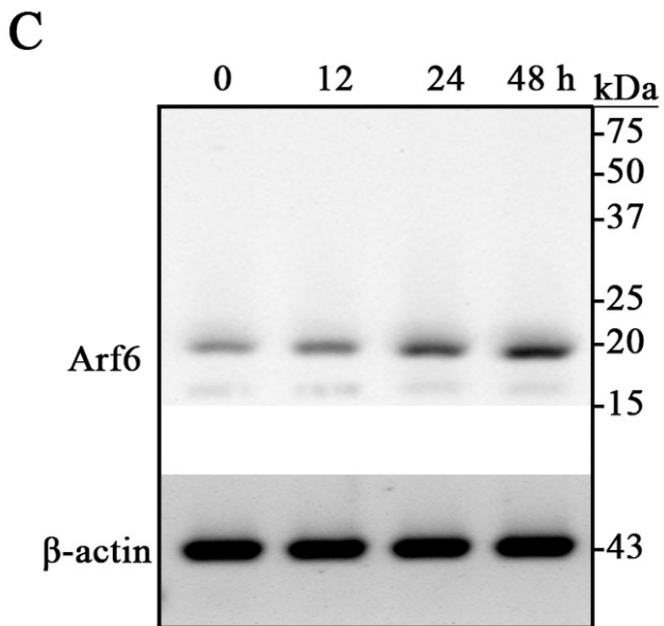

Supplement: S1 Fig — (PDF) [file pone.0229747.s002.pdf]

Supplementary Fig 2. Full length blots of Fig 2A,B

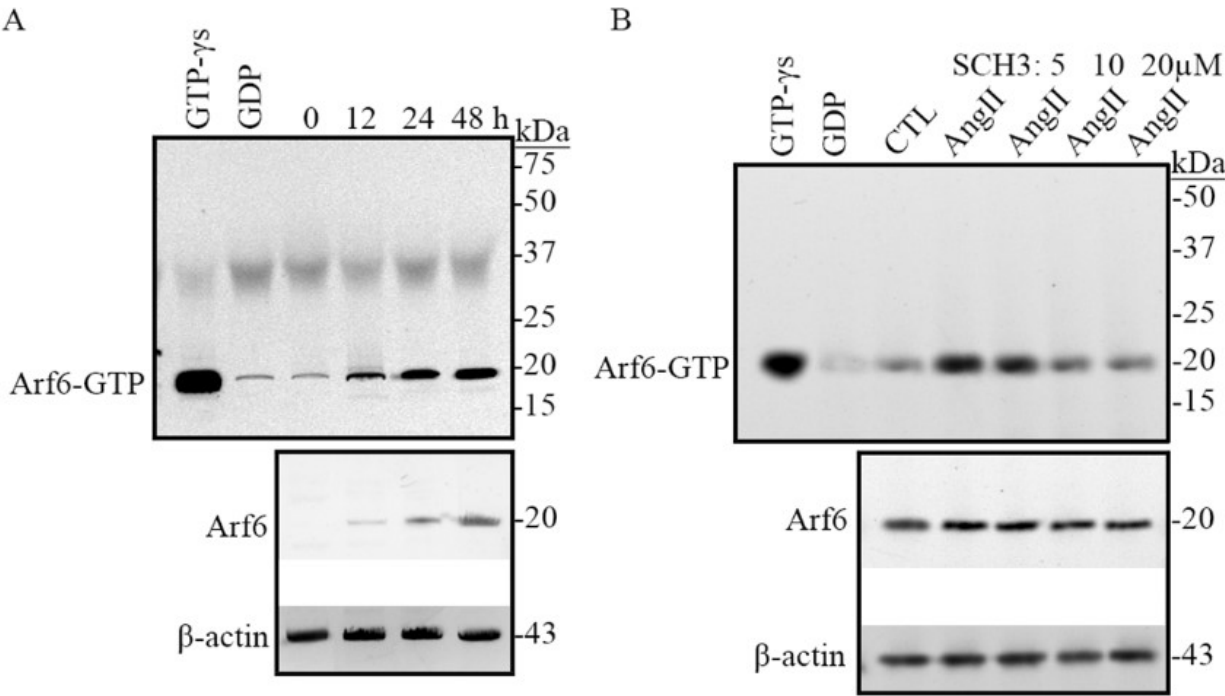

Supplement: S2 Fig — (PDF) [file pone.0229747.s003.pdf]

Supplementary Fig 3. Full length blots of Fig 4A,B,D

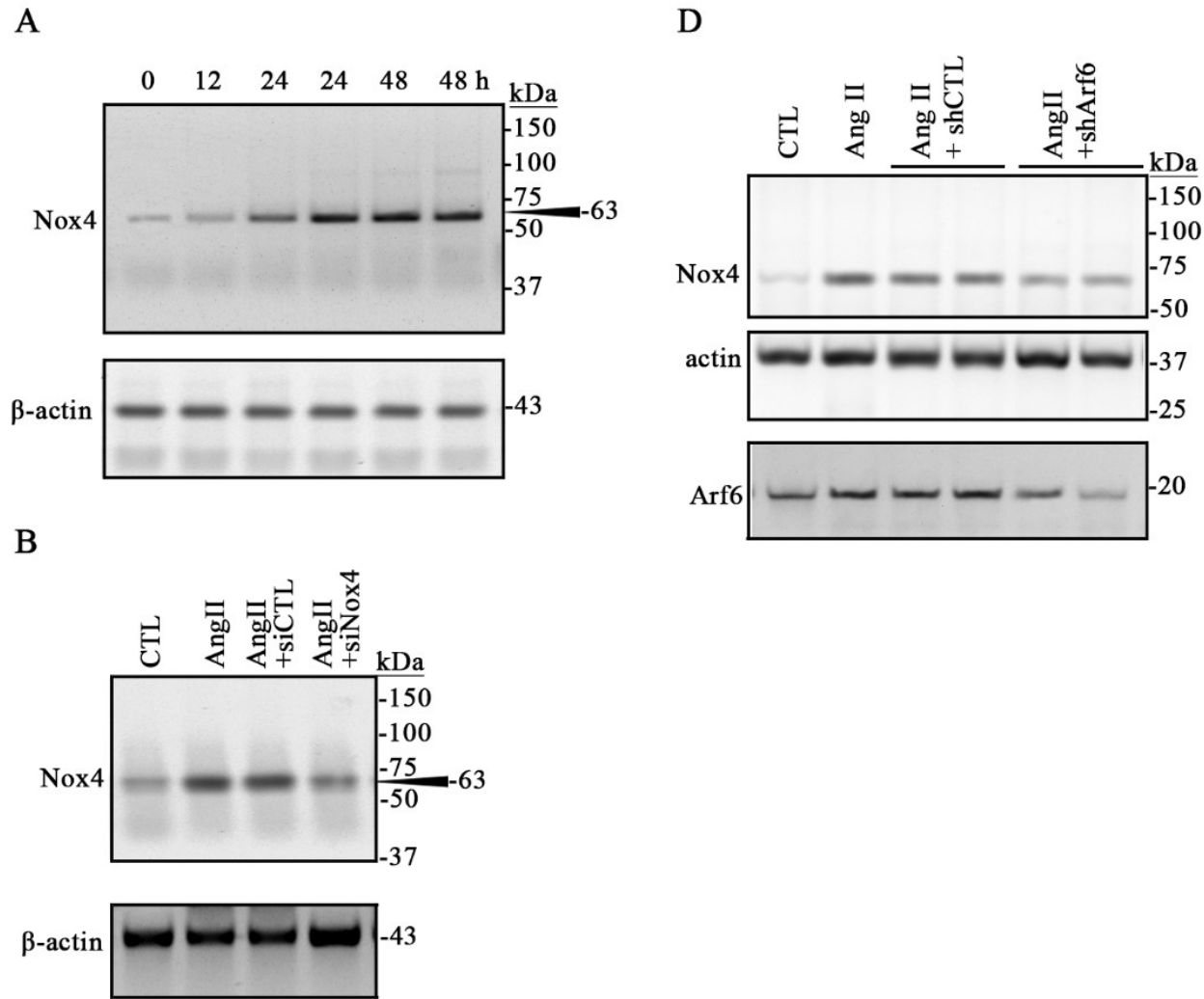

Supplement: S3 Fig — (PDF) [file pone.0229747.s004.pdf]

Supplementary Fig 4. Full length blots of Fig 5A,C,D

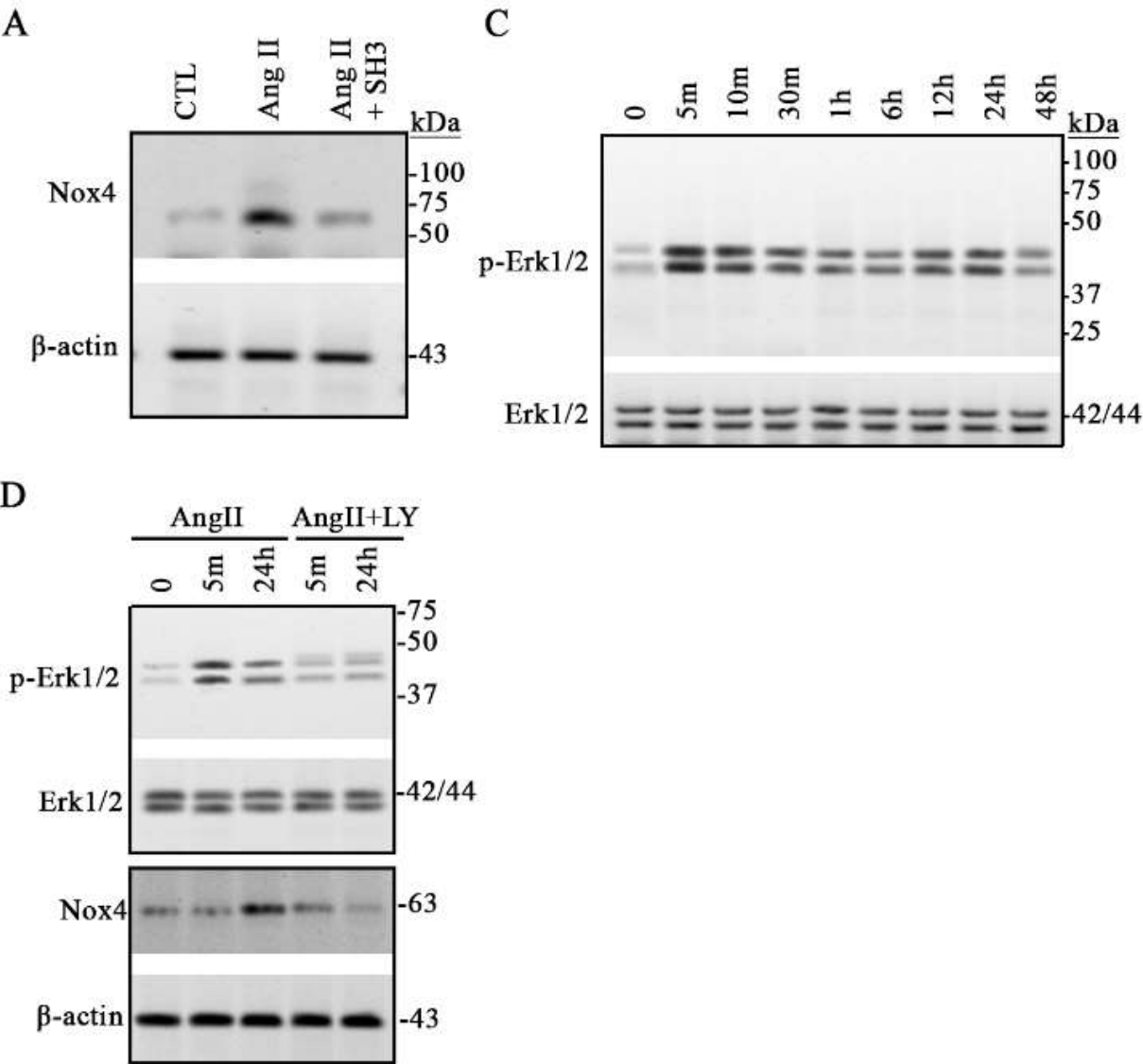

Supplement: S4 Fig — (PDF) [file pone.0229747.s005.pdf]

**Supplementary Fig 5. Full length blots of Fig 6A,B,D**

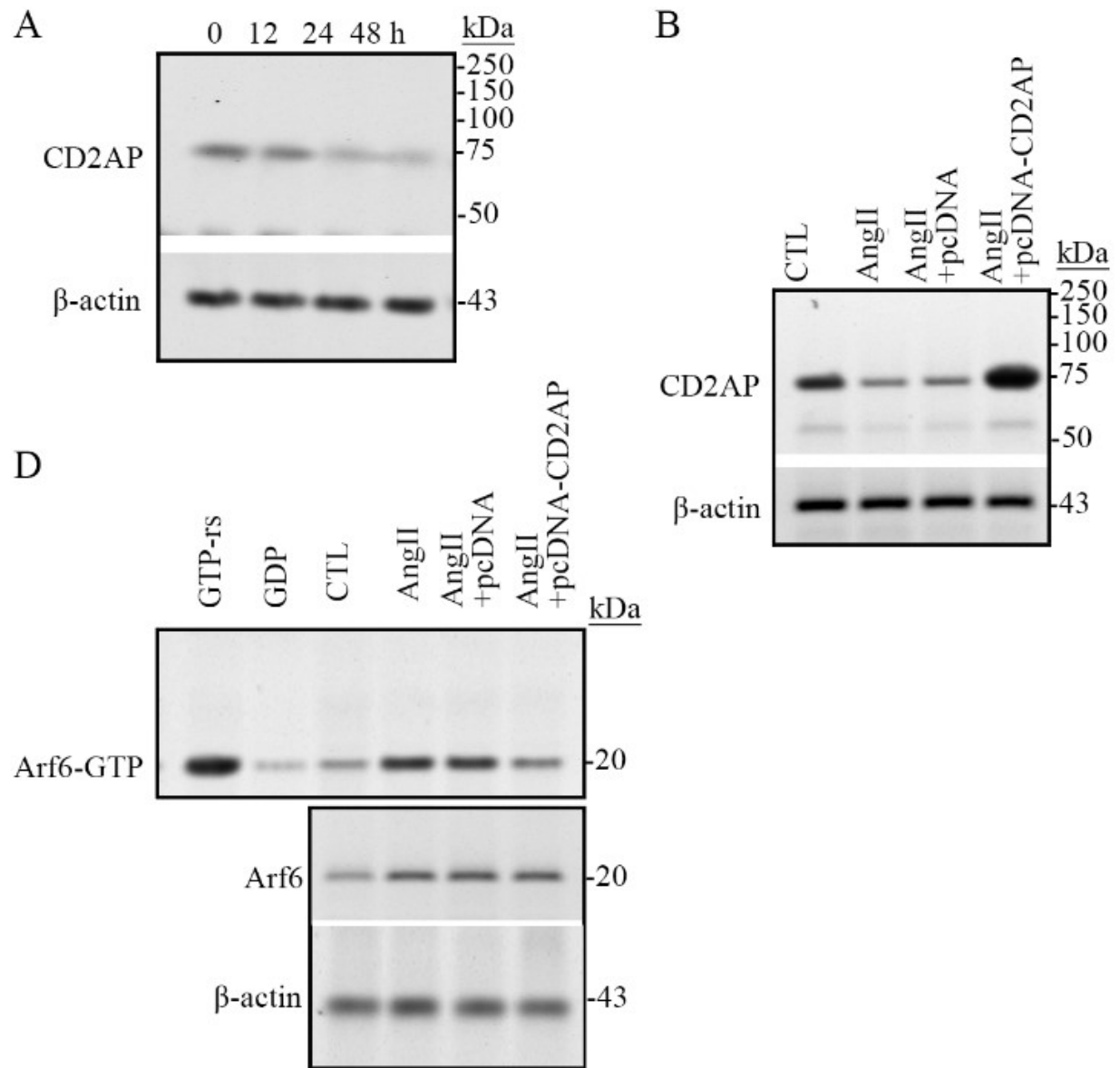

Supplement: S5 Fig — (PDF) [file pone.0229747.s006.pdf]
