# Supplementary material for: Angiotensin II promotes podocyte injury by activating Arf6-Erk1/2-Nox4 signaling pathway
Source: PLoS One. 2020 Mar 2;15(3):e0229747. doi: 10.1371/journal.pone.0229747 (PMC7051060; doi:10.1371/journal.pone.0229747)
Supplement: Attachment — Submitted filename: Response to Reviewers.docx [file pone.0229747.s007.docx]

**We would like to thank the reviewer for reviewing our manuscript and their comments. Point by point responses are outlined below.**

**Reviewer #1:**

In this paper, the authors showed that Ang II increases, via suppression of CD2AP, Arf6 mRNA RNA, protein, and activity in cultured human podocytes. Afr6 enhances Nox4 and ROS production, and then activates caspase 3. Although precise mechanisms underlining Arf6 activation by Ang II were not shown, all data consistently support the authors’ proposal. The major concern is that Ang II does not induce cell death in podocytes in vivo. Moreover, apoptosis is hardly ever observed in podocytes in vivo.

1. The authors should show that the number of dead cells after Ang II stimulation. How much % of podocytes are actually dead dependently on caspase 3. As generally recommended (for example, PMID:17562483), apoptosis should be demonstrated by multiple methods including TUNEL staining. The authors should show whether the effect of Ang II on cell death is blocked by AT1 antagonist and caspase 3 inhibitor.

**Response: As you recommended, the results from TUNEL assay have been provided in the revised manuscript (Fig 2D), showing that the percentage of apoptotic cell death was increased significantly in podocytes treated with Ang II. The data from AT1 antagonist (Losartan) and caspase 3 inhibitor (z-VAD-fmk) were also included, showing that the administration of Losartan and ZAD-fmk significantly reduced Ang II-induced apoptosis.**

2. Active caspase3 is generally stained at perinuclear region, but in Figure 2D, it is overlapped with DAPI staining. There is a concern about specificity of the staining.

**Response: As you recommended, we replaced the active caspase 3 staining with the images from TUNEL assay in the revised manuscript (Fig 2D).**

3. In Fig1D, the staining observed in areas apart from DAPI staining. The authors should present the corresponding phase contrast image to show the location of the Arf6 and morphological change of the cells.

**Response: Arf6 is known to be involved in vesicle trafficking and cellular morphology. It has been shown that Arf6 is also localized both in the cytoplasm and in the cell plasma membrane in podocyte (Lin JS, et al. PLoS ONE. 2017. 12(9): e0184575.), which supports our Arf6 staining pattern. We did not take the phase contrast images; but in the revised manuscript, we provided the blank control staining image (the 2^nd^ antibody only was applied while staining was performed) to show specific staining for Arf6. In addition, the anti-Arf6 antibody that we used is commercial. Therefore, the staining of Arf6 should be specific.**

4. Ang II does not induce apoptosis in podocyte in vivo. The authors should discuss about this discrepancy and biological significance of their findings.

**Response:** **Podocytes are ultimately differentiated glomerular visceral epithelial cells. In proteinuric kidney disease, severe injury results in podocyte differentiation, cell death, detachment, and loss of podocytes.** **Podocyte loss plays a key role in the development of proteinuria and kidney disease. Ang II executes hemodynamic effects on renal tissue, and has a direct influence on induction of podocyte injury by altered expression and distribution of podocyte proteins. Ang II can also promote podocyte injury indirectly by inducing cellular hypertrophy and apoptosis. Some studies have shown that Ang II can result in podocyte apoptosis both *in vitro* and *in vivo*** (Márquez E, et al. Renin-angiotensin system within the diabetic podocyte. Am J Physiol Renal Physiol. 2015;308(1):F1-10; Gao Z, et al. Dab1 Contributes to Angiotensin II-Induced Apoptosis via p38 Signaling Pathway in Podocytes. Biomed Res Int. 2017;2017:2484303; Jia J, Ding G, Zhu J, Chen C, Liang W, Franki N, Singhal PC. Angiotensin II infusion induces nephrin expression changes and podocyte apoptosis. Am J Nephrol. 2008;28(3):500-7; Ren Z, Liang W, Chen C, Yang H, Singhal PC, Ding G. Angiotensin II induces nephrin dephosphorylation and podocyte injury: role of caveolin-1. Cell Signal. 2012;24(2):443-50; Chen X, et al. c-Abl mediates angiotensin II-induced apoptosis in podocytes. J Mol Histol. 2013;44(5):597-608.). **However, the role of apoptotic podocytes in the pathogenesis of proteinuric kidney disease should be fully investigated *in vivo*. Although increasing evidences have been provided showing Ang II can induce podocyte apoptosis through Nox4, this study adds the small GTPase Arf6 to this key cellular event as a novel signaling mechanism. We added this key point and references in the revised manuscript.**

5. The precise role of CD2AP in activation of Arf6 by Ang II is not clear. Possible molecular mechanism should be discussed.

**Response: Yes,** **the precise role and molecular mechanisms by which reduction of CD2AP enhanced Arf6 activation in Ang II-treated podocytes are still not clear.** **We propose that CD2AP may inhibit Arf6 activation possibly by binding to ArfGAPs proteins; but this issue should be further investigated *in vitro* and *in vivo*. We discussed this possible mechanism in the revised manuscript.**

6. In the abstract, “mall” should be “small”.

**Response: We are really sorry for the typing error. We have corrected it, and also checked the entire manuscript carefully for spelling and grammar.**

7. P3, l54, “Angiotensin (Ang II)” should be “Angiotensin II (Ang II)” or “Angiotensin (Ang)

II”

**Response: We have corrected it. Thank you.**

8. The supplier of Ang II should be shown.

**Response: The supplier of Ang II was added in the revised manuscript.**

9. P9, l192, “in the presence of absence of SecinH3” should be “in the presence or absence of

SecinH3”

**Response: We are really sorry for the typing error. We have corrected it in the revised version.**

10. “BV” is an uncommon abbreviation.

**Response: We avoided use of BV, and use the “pcDNA” for blank vector.**

**Reviewer #2:**

In the present manuscript “Angiotensin II promotes podocyte injury by activating Arf6-Erk1/2-Nox4 signaling Pathway”, the authors, Che et al. demonstrated the role of Arf6 in Ang II-induced podocyte injury and shown that Ang II promotes ROS production and apoptosis through activation of Arf6-Erk1/2-Nox4 signaling, in which reduction of CD2AP is responsible for Arf6 activation. This is a well-written paper containing interesting results which merit publication. For the benefit of the reader, however, the authors should consider addressing a few

minor concerns.

1.The authors have used Arf6 inhibitor in their studies to show its role of podocyte injury. However, inhibitor sometimes not so specific because they work by affecting gene expression or activity. Thus, a set of graphs to show the expression of downstream genes by knocking down Arf6 is needed urgently.

**Response: Thank you for your recommendations. We performed knockdown assay with lentiviral shRNA-Arf6 and assessed Nox4 expression and ROS level. These data have been provided in the revised manuscript (Fig 4D,E).**

2.In Figure 1D, the enlarged picture is somewhat fuzzy. Please change the pictures with high resolution image.

**Response: High resolution image has been provided for Fig 1D.**

3.Figure 4, there is something excess in the panel of ß-actin in 4A and Nox4 in 4B. Please confirm the exposure time and treatment groups in the images.

**Response: For unknown reason, sometimes, smear bands were observed in some lanes, likely resulted by an appropriate storage of lysates, unenough boiling, or the lysis buffer itself. Anyway, we replaced it with the samples from the other independent experiment.**

4.Many bar charts were used in this paper. It is kindly suggested to replace it with scatter plot with bar graphs to better represent the number and the dispersion degree of specimens.

**Response: Thank you for your suggestions, we provided the scatter plot images for (****Fig 2D, Fig 3, Fig 4C-E, and Fig 6C,E) in the revised manuscript.**

5.The manuscript is very well written and easy to understand; however, it can use some minor grammatical corrections to improve the flow.

**Response: The revised manuscript has been carefully checked for spelling and grammar.**
