# Supplementary material for: Angiotensin II promotes podocyte injury by activating Arf6-Erk1/2-Nox4 signaling pathway
Source: PLoS One. 2020 Mar 2;15(3):e0229747. doi: 10.1371/journal.pone.0229747 (PMC7051060; doi:10.1371/journal.pone.0229747)
Supplement: Attachment — Submitted filename: Response to Reviewers.docx [file pone.0229747.s008.docx]

**We would like to thank the reviewers for reviewing our manuscript and their comments. Point by point responses are outlined below.**

Reviewer #1:

1. % of dead podocytes and TUNEL should be presented.

The authors added the data of TUNEL staining and showed dependency on caspase 3. Judging from very high rate of TUNEL even in control cells (~10%), the cells the author used were very prone to apoptosis, probably all will die within a few days.

Response: All studies were performed on human podocyte cell line in passages 6-10. For TUNEL assay, cells were cultured on coverslips in 12-well plate and pretreated for 1 h with secinH3 (10 µM), losartan (1 µM) and v-ZAD-fmk (10 µM), respectively. Thereafter, Ang II was applied for 24 h at the concentration of 1 µM. Nontreated cell was used as the controls, but equal amount of DMSO was added because v-ZAD-fmk and secinH3 was dissolved in DMSO. The application of DMSO may have a subtle influence on cellular apoptosis. In addition, we did not see more apoptotic cells for long period culture (e.g. 72 h) in this condition. To make it clear, we added this information and the data (the number of the percentage of apoptotic cell: CTL: 9.1 ± 1.82, Ang II: 27.2 ± 3.98, Ang II+SCH3: 17.5 ± 2.29, Ang II+Los: 6.5 ± 1.57, Ang II+ ZAD 8.0 ± 1.65) in the revised manuscript. We hope that our responses are suitable. Thank you very much.

2. Localization of caspase 3 staining was nuclear.

The authors provided reasonable pictures of caspase 3.

Response: Thank you.

3. Phase contrast pictures would be presented for Fig 1D.

The authors did not provide phase contrast pictures, but this concern is not a central issue.

Response: Thank you.

5. Mechanism of Arf6 inhibition by CD2AP is not clear.

The authors adequately responded by adding a possible mechanism in the discussion.

Response: Thank you.

6-10. Minor errors

The authors adequately amended the errors.

Response: Thank you.

4. Ang II does not induce apoptosis in vivo.

The authors should notice that TUNEL pictures in the reference 23 (Gao Z et al) and 26 (Chen X et al) show many positive staining in tubules without injury nor fragmentation of nuclei, strongly suggesting non-specific staining. Moreover, the EM pictures of ref 24 and 26 do not show apoptosis. These unreliable data are misleading and should not be cited as evidences for in vivo apoptosis. The authors should cite more recent and reliable papers, such as JASN 30: 2307–2320, 2019. doi: https://doi.org/10.1681/ASN.2019010053. This paper demonstrated that Ang II directly exaggerate injury in podocytes of Dynamin knockout mice, in which internalization of AT1R is impaired, but no apoptosis was detected. Judging from the authors’ Figure 5C data, it is speculated that the internalization of AT1R is blunted and Ang II signal is prolonged in the cell line used. The authors should clearly state that there is no reliable evidence that Ang II directly induce apoptosis in podocyte in vivo, and discuss about biological significance of their findings.

Response: Thank you for your recommendations. We removed the references 23, 24 and 26 to avoid misunderstanding and misleading to this key point. About this issue, we discussed as the below:

A large amount of *in vitro* studies has shown that Ang II results in podocyte apoptosis [4-6, 22,23]. Although some studies also show that Ang II can cause podocyte apoptosis in *in vivo* model [22], it should be noted that there is no reliable evidence that Ang II directly induce *in vivo* podocyte apoptosis. The biological significance of apoptosis need be further investigated in Ang II-induced podocyte injury.

Just recently, it has been reported that in dynamin1/2-deficient primary podocytes, Ang II induces abnormal membrane dynamics with increased Rac1 activation and lamellipodial extension, which was attenuated in deficiency of AT1R [32]. This finding suggests that the internalization of AT1R is blunted and Ang II signal is prolonged in podocytes with dynamin1/2 deficiency. Nephrin tyrosine phosphorylation augments Rac1 activity through Arf6, thus leading to focal adhesion turnover and lamellipodial formation in cultured human podocytes [11]. In addition, it has been reported that in cultured podocytes, Ang II treatment results in actin cytoskeleton reorganization, cell adhesion reduction, actin-associated protein downregulation, and albumin permeability increase, in which TRPC6-mediated decrease of MYH9 plays a crucial role [33]. Therefore, actin cytoskeleton remodeling, lamellipodial activity, cell adhesion alteration, and permeability change are the important mechanisms by which Ang II induces structural and functional podocyte injury and thus filtration barrier disruption. The limitation of our study is that we only used apoptosis as the index of podocyte injury. However, our findings reveal a novel function of the small GTPase Arf6 in the context of Ang II induced podocyte injury. Effects of Arf6 activation on actin cytoskeleton, lamellipodial formation, cell adhesion, and permeability should be further evaluated in Ang II treated podocytes.
